# Access to Rehabilitation After Hospitalization for Traumatic Brain Injury: A National Longitudinal Cohort Study in Sweden

**DOI:** 10.1177/15459683231209315

**Published:** 2023-11-12

**Authors:** Andrea Klang, Yasmina Molero, Paul Lichtenstein, Henrik Larsson, Brian Matthew D’Onofrio, Niklas Marklund, Christian Oldenburg, Elham Rostami

**Affiliations:** 1 Department of Medical Sciences, Rehabilitation Medicine, Uppsala University, Uppsala, Sweden; 2 Department of Clinical Neuroscience, Karolinska Institutet Stockholm, Sweden; 3 Department of Medical Epidemiology and Biostatistics, Karolinska Institutet, Stockholm, Sweden; 4 Department of Medical Sciences, Örebro University, Örebro, Sweden; 5 Department of Psychological and Brain Sciences, Indiana University, Bloomington, IN, USA; 6 Department of Medical Sciences, Neurosurgery, Uppsala University, Uppsala, Sweden; 7 Department of Clinical Neuroscience, Karolinska Institutet, Stockholm, Sweden; 8 Department of Neuroscience, Karolinska Institutet, Stockholm, Sweden; 9 Department of Clinical Sciences Lund, Neurosurgery, Lund University, Skåne University Hospital, Lund, Sweden

**Keywords:** traumatic brain injury, neurorehabilitation, rehabilitation, brain injury, traumatic, brain trauma

## Abstract

**Background:**

Rehabilitation is suggested to improve outcomes following traumatic brain injury (TBI), however, the extent of access to rehabilitation among TBI patients remains unclear.

**Objective:**

To examine the level of access to rehabilitation after TBI, and its association with health and sociodemographic factors.

**Method:**

We conducted a longitudinal cohort study using Swedish nationwide healthcare and sociodemographic registers. We identified 15 880 TBI patients ≥18 years hospitalized ≥3 days from 2008 to 2012 who were stratified into 3 severity groups; grade I (n = 1366; most severe), grade II (n = 5228), and grade III (n = 9268; least severe). We examined registered contacts with specialized rehabilitation or geriatric care (for patients ≥65 years) during the hospital stay, and/or within 1 year post-discharge. We performed a generalized linear model analysis to estimate the risk ratio (RR) for receiving specialized rehabilitation or geriatric care after a TBI based on sociodemographic and health factors.

**Results:**

Among TBI patients, 46/35% (grade I), 14/40% (grade II), and 5/18% (grade III) received specialized rehabilitation or geriatric care, respectively. Being currently employed or studying was positively associated (RR 1.7, 2.3), while living outside of a city area was negatively associated (RR 0.36, 0.79) with receiving specialized rehabilitation or geriatric care. Older age and a prior substance use disorder were negatively associated with receiving specialized rehabilitation (RR 0.51 and 0.81).

**Conclusion:**

Our results suggest insufficient and unequal access to rehabilitation for TBI patients, highlighting the importance of organizing and standardizing post-TBI rehabilitation to meet the needs of patients, regardless of their age, socioeconomic status, or living area.

## Introduction

Traumatic brain injury (TBI) is a major health problem and a leading cause of disability and mortality world-wide.^1^ The incidence of hospital admissions due to TBI is reported to be around 250 to 300 cases per 100 000 individuals.^2,3^ Primary prevention such as improved car and road safety and fall prevention amongst the elderly is the most effective measure to decrease the disease burden.^4^ Additionally, long-term effects of TBI can be reduced by minimizing secondary insults through optimal acute care (ie, neurosurgical surveillance and neurocritical care).^5^ However, a significant proportion of individuals will still suffer from disabling symptoms after a TBI, ranging from headaches and fatigue to paresis and severe cognitive and behavioral problems.^6^ The majority of individuals with severe TBI will have some degree of long-lasting symptoms and disability,^7-9^ however, persistent symptoms are common regardless of the severity of the TBI.^10-13^

Studies suggest that early rehabilitation after a severe TBI can improve outcomes^14^ and be more cost-effective than delayed interventions.^15^ Despite the known impact of rehabilitation on outcomes,^16,17^ not all TBI patients are offered rehabilitation and access differs between countries:^18^ In a French study, only 45% received rehabilitation after severe TBI.^19^ The corresponding number in the Netherlands was 50%,^20^ and in Norway 75%.^21^ However, these studies have either been restricted to a small geographical area,^19,20^ or only included a small number of patients per country,^6,18^ affecting the generalizability of results. This underscores the importance of examining the access to rehabilitation after a TBI in a nationwide context.

Several sociodemographic factors have been associated with access to rehabilitation after a TBI.^18^ Studies consistently show that younger individuals and males are more likely to receive rehabilitation after a TBI.^9,18,19^ Having a higher level of education, being employed or studying also increases the likelihood of receiving specialized rehabilitation.^18,21,22^ However, factors such as premorbid psychiatric disorders or living area have not been studied in relation to post-TBI rehabilitation. Psychiatric problems have been shown to impede access to other areas of healthcare^23,24^ and similarly, a rural setting has been linked to lower access to, for example, stroke rehabilitation.^25,26^ In order to improve access to rehabilitation, hindering factors need to be identified.

As a majority of previous studies have only included patients treated for TBI in neurotrauma centers, little is known about the access to rehabilitation for the large number of patients who are never referred to a tertiary hospital, such as patients with less severe TBIs. Furthermore, studies of TBI cohorts commonly have high rates of loss to follow up,^27,28^ and TBI severity and sociodemographic factors have been associated with a further increased risk of attrition from studies. Nationwide patient registers have the advantage of following up all patients regardless of where they are treated or willingness to participate in follow-up studies. This knowledge is crucial for developing national strategies and guidelines that ensure a high and equal access to rehabilitation.

The aim of this study was to examine access to rehabilitation in a Swedish nationwide context during the first year following a TBI. Secondly, we aimed to investigate whether access to rehabilitation was associated with health and sociodemographic factors.

## Method

### Ethics

The project follows the Declaration of Helsinki and was approved by the Swedish Ethical Review Authority (2013/5:8), which waived the need for informed consent due to the register-based design. All data were anonymized.

### Design

This is a population-based longitudinal study in Sweden, linking several nationwide high-quality registers, including the Swedish Patient Register,^29^ the Cause of Death Register,^30^ the Total Population Register,^29^ and the Longitudinal Integrated Database for Health Insurance and Labour Market Studies (LISA).^29^

### Study Population

Our cohort included all individuals who were hospitalized between 1 January 2008 and 31 December 2012 with TBI registered as the main diagnosis, and who were age 18 years or older on the day of their injury. We excluded patients with a TBI diagnosis during 2007 to avoid readmissions for the same TBI. Because we wanted to examine patients with the need for prolonged observation and/or treatment, we excluded patients who had been hospitalized for less than 3 days.

We used the Centers for Disease Control and Prevention definition. This included the International Classification of Diseases, 10th revision (ICD-10) codes: S02.0, S02.1, S02.3, S02.7 to S02.9, S04.0, S06.0 to S06.9, S07.0, S07.1, S07.8. S07.9, S09.7 to S09.9, excluding open wounds and sequelae: S01, T01.0, and T90. Information was extracted from the National Patient Register, which has a sensitivity ranging between 95% and 97%; and a specificity ranging between 96% and 98% for TBI diagnoses received during inpatient treatment.^31^

In order to address the severity of TBI, patients were classified into 3 hierarchical and mutually exclusive groups in the following order: (1) grade I TBI, that is, patients who underwent a neurosurgical procedure;^32^ (2) grade II TBI, that is, patients without neurosurgical procedure with a hospital stay of 10 days or more, and; (3) grade III TBI, patients without neurosurgical procedure with a hospital stay of 3 to 9 days.

#### Sociodemographic Data

Information on sociodemographic data was collected from LISA as registered in November in the year prior to the TBI and included the highest completed level of education, source of income, and civil status (married, divorced/widowed, unmarried). Age at TBI (in years), sex (female or male), and country of birth (Sweden, Europe, and outside of Europe) were collected from the Total Population Register. Municipal residency was collected from LISA as registered in November in the year before the TBI and was categorized according to Eurostat’s classification of degree of urbanization, DEGURBA,^33,34^ which contains 3 levels; city, town/suburban area, and rural area, for details see Supplemental Appendix.

#### Premorbidities

Information on lifetime prevalence of neurological and psychiatric disorders before the date of the TBI was collected from the National Patient Register, and included dementia, other neurodegenerative disorders, stroke, substance use disorders, and other psychiatric disorders, for ICD-10 codes see Supplemental Appendix.

#### Specialized Rehabilitation

In Sweden, specialized rehabilitation after TBI is provided by departments of rehabilitation medicine.^35^ Multidisciplinary teams with experience of acquired brain injury make assessments of remaining symptoms and impact of function, and set up an individualized plan of rehabilitation. We defined access to specialized rehabilitation as the presence of a registered contact with a rehabilitation medicine department (including neurological rehabilitation) either during the initial TBI treatment (ie, between hospital admission for the TBI until discharge), and/or post-TBI treatment (ie, within 365 days after hospital discharge). Post-TBI treatment included both inpatient and outpatient contacts.

#### Geriatric Care

For older patients (≥65 years), multidisciplinary rehabilitation can also be provided by departments of geriatric care and rehabilitation.^35,36^ We defined access to geriatric care as the presence of a registered contact with a geriatric care department during the initial TBI treatment and/or post-TBI treatment (ie, within 365 days after hospital discharge) for patients ≥65 years. Post-TBI treatment included both inpatient and outpatient contacts.

#### Other Care and Co-Occurring Injuries

We also identified registered contacts with other medical departments during the initial TBI treatment (ie, between hospital admission for the TBI until discharge), including; neurosurgical, non-neurosurgical with experience of neurological monitoring (ie, surgery, internal medicine, neurology, and stroke), orthopedic, and other (ie, all other departments).

Co-occurring injury was defined as spinal cord injuries, fractures, or other injuries (excluding TBI diagnoses) during the initial hospital stay, for ICD-10 codes see Supplemental Appendix.

### Statistical Analyses

The analyses of the TBI cohort are presented separately for each of the 3 TBI groups, that is, grade I, grade II, and grade III TBI. For each TBI group, we calculated the proportion of patients who had been treated at a neurosurgical, non-neurosurgical, orthopedic, geriatric, rehabilitation medicine, and/or other department during their initial hospital stay (ie, between initial admission for the TBI and the day of hospital discharge). We also calculated the median length of stay (including the interquartile range [IQR]) within each department, defined as the number of days between admission and discharge within that department. We then calculated the proportion of patients with post-discharge care within specialized rehabilitation or geriatric care during the initial hospital stay and/or within 365 days after hospital discharge.

We performed a generalized linear model (GLM) analysis to estimate the risk ratio (RR) of receiving specialized rehabilitation or geriatric care (yes/no), respectively, based on TBI group membership (grades I-III). We ran the PROC GENMOD procedure in SAS using a Poisson distribution with a robust variance estimator and log link function. Each of the 3 TBI groups was treated as a class exposure, with grade III (representing the lowest TBI severity) serving as the reference group for comparisons against grade I and grade II. We estimated the RR of receiving specialized rehabilitation or geriatric care based on TBI group membership in 3 steps to consider potential confounders: First, we ran an unadjusted analysis where we calculated the RR of receiving specialized rehabilitation or geriatric care without any adjustments. Second, we adjusted the analysis for increasing age and male sex. Third, we performed an analysis that adjusted for multiple factors, including sex, age, sociodemographic factors (living area, education, and source of income), premorbidities (substance use disorders, psychiatric disorders, neurodegenerative diseases, and stroke), and TBI-related factors (co-occurring injuries).

We then stratified the TBI cohort into 3 groups; grade I, grade II and grade III, and examined associations between health and sociodemographic factors and access to specialized rehabilitation or geriatric care within each group. We performed GLM analyses to estimate the RR of receiving specialized rehabilitation or geriatric care for each health and sociodemographic factor separately, including sex (females compared to males), living area (living in a rural or town/suburban area compared to living in a city), education level (>12 years of education or upper secondary education compared to primary education), being employed and/or studying (compared to being unemployed and/or retired), lifetime prevalence of substance use disorder (compared to no diagnosis), lifetime prevalence of psychiatric disorder (compared to no diagnosis), lifetime prevalence of neurodegenerative disorder (compared to no diagnosis), lifetime prevalence of stroke (compared to no diagnosis), and co-occurring injury (compared to no other injury),

#### Sensitivity Analyses

We conducted sensitivity analyses where we examined only patients who were under age 65 at the time of their TBI, since many departments of rehabilitation medicine may only include patients of working age. First, we performed GLM analysis to estimate the RR of receiving specialized rehabilitation in those under 65 years based on TBI group membership (grades I-III). This was done in 3 steps: (1) unadjusted analysis; (2) analysis adjusted for increasing age and male sex, and; (3) analysis adjusted for multiple factors (as defined above). We then stratified TBI patients under age 65 years into 3 groups (grades I-III) and examined associations between health and sociodemographic factors and access to specialized rehabilitation within each group (in the same manner as described in the main analyses).

RRs and 95% confidence intervals [CIs] are presented for all GLM analyses. We used SAS version 9.4. The Strengthening the Reporting of Observational studies in Epidemiology (STROBE) reporting guidelines were followed.^37^

## Results

### TBI Cohort Selection

Our final cohort included 15 880 individuals with TBI. We initially identified 163 810 individuals who had been treated for a TBI in a hospital or specialized open care between 1 January 2008 and 31 December 2012. We then excluded individuals who; (a) had been treated for a TBI in 2007, (b) were under age 18 years, (c) had died before discharge, (d) were not hospitalized or hospitalized for less than 3 days, and (e) were not diagnosed with TBI as a primary diagnosis. Of the 15 880 individuals in the cohort, 9% (n = 1366) fit the criteria for a grade I TBI (ie, received a neurosurgical procedure), 33% (n = 5228) for a grade II TBI (ie, inpatient treatment for 10 days or more without a neurosurgical procedure), and 58% (n = 9286) for a grade III TBI (ie, inpatient treatment for 3 to 10 days without a neurosurgical procedure). For a complete flow chart, see Supplemental Appendix Figure A.

### Demographic and Health Characteristics of the Cohort

Median age at TBI was 55 (IQR 36-67) in the grade I group, 79 (IQR 65-86) in the grade II group, and 74 years old (IQR 58-84) in the grade III group. In the grade II and grade III groups, the majority of patients (76% and 64%) were 65 years or older at the time of the TBI. In the grade I group, 26% were women, while near half of the grade II and III groups were women (45% and 44%). More than half of the patients with grade I TBI were either currently employed (43%) or studying (8%), while pension was the most common source of income among grade II and III groups (77% and 67%). Living area, that is, living in a city, town/suburban, or rural area, was evenly distributed across the 3 groups. For more details on demographic factors, see Table 1.

**Table 1.** Baseline of Premorbid Demographic and Health Characteristics of the Groups.

	Grade I TBI (n = 1366)	Grade II TBI (n = 5228)	Grade III TBI (n = 9286)
Age at TBI (y)			
18-34	23.7% (323)	4.9% (257)	8.7% (807)
35-49	18.0% (246)	5.8% (305)	8.8% (816)
50-64	29.5% (403)	13.4% (699)	17.0% (1585)
65-79	23.1% (316)	28.3% (1477)	28.4% (2639)
80+	5.71% (78)	47.6% (2490)	37.0% (2439)
Median age (IQR)	55 (36, 67)	79 (65, 86)	74 (58, 84)
Sex			
Females	25.6% (350)	44.8% (2344)	43.7% (4059)
Males	74.4% (1016)	55.2% (2844)	56.3% (5227)
Civil status^a^			
Married	31.6% (431)	35.6% (1862)	36.9% (3424)
Divorced or widowed	23.9% (326)	47.1% (2461)	40.6% (3766)
Unmarried	44.1% (602)	17.0% (888)	22.2% (2064)
Occupation			
Employed	43.5% (594)	16.3% (850)	23.2% (2158)
Study income	8.5% (116)	1.7% (88)	2.8% (258)
Pension	32.0% (436)	77.0% (4025)	67.4% (6258)
Disability pension	12.8% (175)	6.8% (356)	8.2% (760)
Country of birth^b^			
Sweden	87.3% (1192)	89.5% (4679)	88.6% (8231)
Europe (excl. Sweden)	8.9% (122)	8.5% (445)	8.6% (799)
Outside of Europe	3.8% (52)	2.0% (104)	2.8% (255)
Education^c^ (y)			
0-9	32.6% (445)	45.2% (2362)	42.0% (3897)
10-12	47.0% (642)	36.5% (1906)	38.0% (3532)
>12	18.6% (254)	15.9% (833)	17.9% (1662)
Degree of urbanization—DEGURBA^d^			
Cities	35.3% (482)	39.6% (2071)	34.5% (3219)
Towns and suburbs	30.4% (415)	30.8% (1611)	32.4% (3004)
Rural areas	33.8% (462)	29.2% (1527)	32.6% (3027)
Premorbid diagnoses			
Substance use disorders	18.5% (252)	8.6% (451)	9.7% (898)
Psychiatric disorders	15.8% (216)	13.4% (698)	13.8% (1286)
Dementia	1.0% (13)	5.7% (299)	5.7% (525)
Other neurodegenerative disorder	1.8% (24)	6.0% (314)	5.2% (480)
Stroke	6.2% (85)	14.8% (776)	11.9% (1105)
Co-occurring injuries			
Fractures	26.0% (355)	21.0% (1094)	12.6% (1174)
Spinal cord injuries	1.0% (13)	0.3% (15)	0.1% (5)
Other injuries	28.4% (388)	20.0% (1042)	18.7% (1733)

Occupation categories are not mutually exclusive. Grade I = neurosurgical intervention, grade II ≥ 10 days hospitalization, and grade III = 3 to 10 days hospitalization.

a Missing information on 56 individuals.

b Missing information on 1 individual.

c Missing information on 347 individuals.

d Missing information on 62 individuals.

The most common premorbid diagnosis (ie, before the TBI) was substance use disorder amongst individuals with grade I TBI (18%). For the grade II and III groups, stroke (15%) and psychiatric disorders (14%), respectively, were the most common disorders. The grade I group had a higher rate of co-occurring injuries (ie, that co-occurred with the TBI), including fractures (26%) and other injuries such as wounds and soft tissue injuries (28%). Spinal cord injuries were rare in all TBI groups (1% or less).

## Initial TBI Treatment

The median time of hospital stay during the initial TBI treatment was 25 days (IQR 11-54) for the grade I group, 16 days (IQR 12-25) for grade II, and 5 days (IQR 3-7) for grade III. The majority of patients with grade I TBI were treated at a neurosurgical department (89%), compared to grade II and III that were mainly treated at non-neurosurgical departments with experience of neurological monitoring (ie, general surgery, internal medicine, neurology, and stroke). For detailed information of clinical departments, time of stay and complications, see Supplemental Appendix Table A.

### Access to Specialized Rehabilitation and Geriatric Care

**Figure 1.**
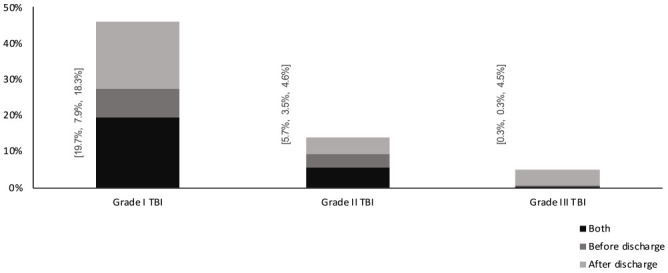
Inpatient and/or outpatient specialized rehabilitation within 365 post TBI; during initial hospital stay, after discharge or during both initial hospital stay, and after discharge. *Note.* Grade I = neurosurgical intervention, grade II ≥ 10 days hospitalization, and grade III = 3 to 10 days hospitalization.

Almost half of the patients in the grade I group (46%; n = 627) received specialized rehabilitation during the initial hospital stay and/or within 365 days post discharge. The corresponding rate for grade II and III was 14% (n = 726) and 5% (n = 475). For details see Figure 1.

**Figure 2.**
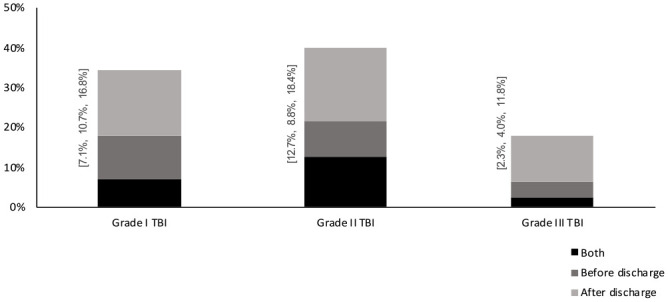
Inpatient and/or outpatient geriatric care and rehabilitation within 365 post TBI; during initial hospital stay, after discharge or during both initial hospital stay, and after discharge for individuals ≥65 years. Grade I TBI n = 395; Grade II TBI n = 3967; and Grade III TBI n = 6078. *Note.* Grade I = neurosurgical intervention, grade II ≥ 10 days hospitalization, and grade III = 3 to 10 days.

Two-thirds of the patients in our cohort were 65 years or older (n = 10 439), and we examined the prevalence of geriatric care in this age group specifically. Results showed that 35% of those in grade I, 40% in grade II and 18% in grade III, received geriatric care at some point during the first year, see Figure 2.

We then performed a GLM analysis to estimate the RR of receiving specialized rehabilitation or geriatric care based on TBI group membership. We compared patients in the grade I to grade III, and found that grade I patients demonstrated an increased RR (9.06 [95% CI 8.16, 10.06]) of receiving specialized rehabilitation. The RR decreased when adjusting for increasing age and male sex (4.83 [4.34, 5.38]), and remained unchanged after adjustments for multiple health and sociodemographic factors. When we compared grade II to grade III patients, we found an increased RR (2.71 [2.42, 3.03]). The RR increased after adjusting for increasing age and male sex (3.45 [3.11, 3.84]) and remained similar during further adjustments. Grade I patients were also more likely to receive geriatric care than grade III patients (1.92 [1.66, 2.22]), with only small changes after further adjustments. Similarly, the RR of receiving geriatric care increased for grade II patients as compared to grade III (2.22 [2.08, 2.37]), with marginal changes in the RR after further adjustments, see Table 2.

**Table 2.** Relative Risk Ratio for Receiving Specialized Rehabilitation or Geriatric Rehabilitation and Care [95% CI].

	Specialized rehabilitation for patients all ages	Geriatric care and rehabilitation for patients ≥65 y
Unadjusted^a^		
Grade I	9.06 [8.16, 10.06]	1.92 [1.66, 2.22]
Grade II	2.71 [2.42, 3.03]	2.22 [2.08, 2.37]
Adjusted for sex and age^a^		
Grade I	4.83 [4.34, 5.38]	2.14 [1.85, 2.49]
Grade II	3.45 [3.11, 3.84]	2.19 [2.05, 2.33]
Full adjustment^a,b^		
Grade I	4.84 [4.35, 5.37]	2.23 [1.93, 2.58]
Grade II	3.52 [3.18, 3.91]	2.08 [1.95, 2.22]

Grade I = neurosurgical intervention, grade II ≥ 10 days hospitalization, and grade III = 3 to 10 days hospitalization.

a Reference TBI Grade III.

b Adjusted for male sex, age, degree of urbanization, level of education, source of income, co-occurring injuries and premorbid: substance use disorder, psychiatric disorder, neurodegenerative disease, and stroke.

### Health and Sociodemographic Predictors of Specialized Rehabilitation and Geriatric Care

**Figure 3.**
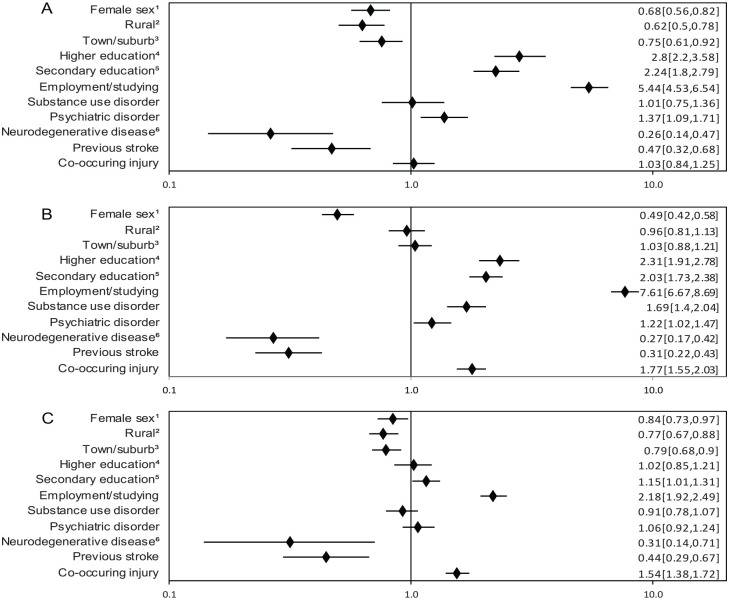
(A) Predictors of specialized rehabilitation within 365 days after Grade I TBI. Risk ratio (RR; adjusted 95% CI). (B) Predictors of specialized rehabilitation within 365 days after Grade II TBI. RR (adjusted 95% CI). (C) Predictors of specialized rehabilitation within 365 days after Grade III TBI. RR (adjusted 95% CI). ^1^Reference male sex. ^2^Living in rural area (DEGURBA). Reference City area. ^3^Living in town/suburban area (DEGURBA). Reference City area. ^4^>12 y of education. Reference primary education <9 y. ^5^10-12 y of education. Reference primary education <9 y. ^6^ Including dementia Missing information for the total TBI-cohort: DEGURBA for 62 individuals, level of education for 56 individuals, occupation for 347 individuals.

Patients with grade I TBI who lived in a rural (RR 0.63 [0.50, 0.78]) or town/suburban area (0.75 [0.61, 0.92]) were less likely to receive specialized rehabilitation, compared to those living in a city. Similar associations were seen for grade III TBI (0.77 [0.67, 0.88] and 0.79 [0.68, 0.90]). Being currently employed or studying was positively associated with receiving specialized rehabilitation in all groups (5.44 [4.53, 6.54]; 7.61 [6.67, 8.69]; and 2.18 [1.92, 2.49]). We found a positive association between level of education receiving specialized rehabilitation in grade I and II, with the highest RR for higher education (2.8 [2.2, 3.58] and 2.31 [1.91, 2.78]). Females were less likely to receive specialized rehabilitation in any of the 3 groups (0.68 [0.56, 0.82]; 0.49 [0.42, 0.58]; and 0.84 [0.73, 0.97]). The presence of co-occurring injuries was positively associated with receiving specialized rehabilitation in grade II and grade III (1.77 [1.55, 2.03] and 1.54 [1.38, 1.72]). We found no consistent associations between premorbid diagnoses and rehabilitation. For details, see Figure 3A to C.

**Figure 4.**
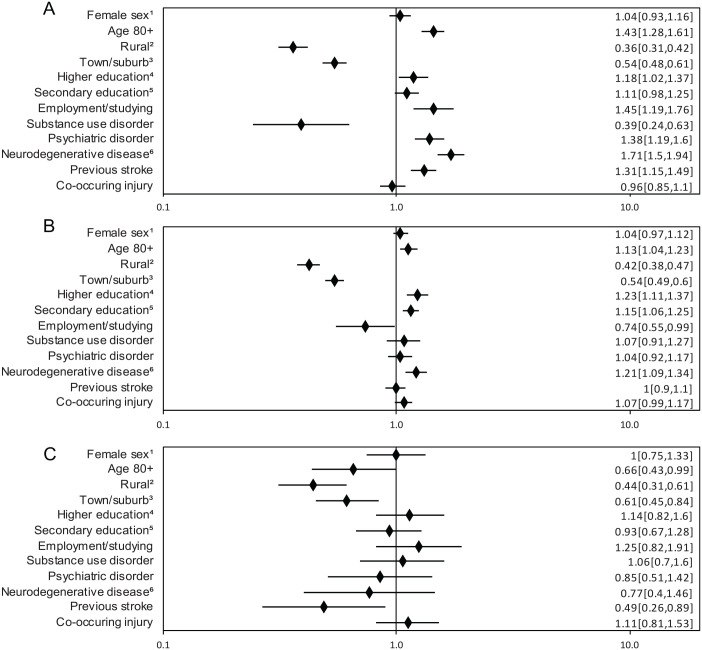
(A) Predictors of geriatric care after Grade I TBI for patients ≥65 years. Risk ratio (RR; adjusted 95% CI). (B) Predictors of geriatric care after Grade II TBI for patients ≥65 years. RR (adjusted 95% CI). (C) Predictors of geriatric care after Grade III TBI for patients ≥65 years. RR (adjusted 95% CI). ^1^Reference male sex. ^2^Living in rural area (DEGURBA). Reference City area. ^3^Living in town/suburban area (DEGURBA). Reference City area. ^4^>12 y of education. Reference primary education <9 y. ^5^10-12 y of education. Reference primary education <9 y. ^6^Including dementia Missing information for the total TBI-cohort: DEGURBA for 62 individuals, level of education for 56 individuals, occupation for 347 individuals.

Patients aged 65 year or older were more likely to receive geriatric care if they were living in a city, regardless of the severity of the TBI. There was no consistent assocation between sex, age, level of education, comorbidites and access to geriatric care. For RRs and CIs, see Figure 4A to C.

### Sensitivity Analyses

In sensitivity analyses, we included only patients of working age (<65 years). We ran a GLM analysis to estimate the RR of receiving specialized rehabilitation based on TBI group membership and found in the unadjusted analyses that both grade I and grade II patients were more likely to receive specialized rehabilitation (4.84 [4.36, 5.38] and 3.67 [3.29, 4.10]). Associations remained similar after further adjustments. For details see Supplemental Appendix Table B.

We then examined each TBI group separately, presented in Supplemental Appendix Figures B1 to 3. Having a current employment and/or studying remained positively associated with receiving specialized rehabilitation in all 3 TBI groups (1.6 [1.28, 1.98]; 1.57 [1.35, 1.82]; and 1.3 [1.15, 1.47]). Associations between level of education and specialized rehabilitation remained for grade I (secondary education: 1.36 [1.07, 1.73]; higher education: 1.82 [1.40, 2.36]); and grade II (higher education: 1.22 [1.03, 1.45]). Being between 35 to 49 and 50 to 64 years of age at the time of the TBI was negatively associated with receiving specialized rehabilitation (for RRs see Supplemental Appendix Figure B1-B3) for all severities, as compared to being 18 to 34 years. Substance use disorder was negatively associated with receiving specialized rehabilitation in all 3 groups (for RRs see Supplemental Appendix Figure B1-B3). We found no associations for sex.

## Discussion

This study included 15 880 individuals who had been hospitalized for a TBI for at least 3 days between 2008 and 2012 and is, to our knowledge, the first nationwide study to examine access to rehabilitation after a TBI. We found that 46% had received specialized rehabilitation within the first year after a grade I TBI (ie, most severe), while 14% of those with grade II and 5% of those with grade III (ie, least severe) TBI patients had received specialized rehabilitation. For TBI patients over age 65 years, the corresponding rates for geriatric care was 35% (grade I), 40% (grade II), and 18% (grade III). We found that younger age and having a current employment or studying were most strongly associated with receiving specialized care after a TBI. Living outside of a city and having a prior substance use disorder (among patients <65 years) was negatively associated with receiving specialized rehabilitation or geriatric care.

The overall access to specialized rehabilitation was low for all severities of hospitalized TBI, in line with several other European studies.^6,18^ Grade III included the largest group of patients in our cohort (around 60%), and also had the lowest prevalence rate of specialized rehabilitation. This could affect relative RRs in our comparisons of grade III against grade I and grade II. Furthermore, because grade III represented patients with less severe TBIs, the need for rehabilitation could be expected to be lower in this group. However, recent data show that a large majority of patients who have been hospitalized with mild TBI have disabling symptoms 6 months after the injury^6^ and it raises the question if some patients need for rehabilitation is unmet. Rehabilitation rates were lower as compared to our neighboring Scandinavian countries Denmark and Norway, where rates of specialized rehabilitation after a severe TBI are 84% and 75%, respectively^21,22^ although these studies classified severe TBI by the Glasgow Coma Scale at admission. Denmark has a centralized specialized rehabilitation at 2 hospitals since 2000,^22^ which could explain the higher rates of access to specialized rehabilitation after a TBI as compared to Sweden. Multidisciplinary support and care following a TBI has demonstrated benefits,^38^ and limited access to rehabilitation could increase the burden for the patient to cope and adapt to the new circumstances.^39,40^ This could particularly affect patients with a large impact on their level of function; such as patients with cognitive and speech problems (which are common after a TBI) who might have difficulties in conveying their needs to healthcare.^6^ Limited access to rehabilitation after a TBI could also add an extra strain on the surrounding family and social support,^39,40^ who are then forced to navigate the healthcare system and domestic services on their own.^38,39^

The high age in our TBI cohort, where the majority of grade II and grade III patients were 65 years or older, is in line with the paradigm shift of TBI, with an increased incidence amongst the elderly^3,41^. This change is probably multifactorial and could be attributed to higher life expectancy and increased activity among the elderly resulting in more accidents.^3,41^ Age is not a disqualification for access to specialized rehabilitation, yet, even among patients of working age, higher age was negatively associated with receiving specialized rehabilitation in our study, as suggested in prior studies.^9,22,42^ Rehabilitation of older patients with TBI may be better provided by other facilities (eg, geriatric care) given that appropriate care is provided. However, our results showed that the access to geriatric care was low across all severities (18%-40%). Newly added symptoms, especially more invisible disabilities such as memory impairment or concentration problems, may be regarded as a result of aging and other comorbidities, by both healthcare professionals and the patients. Physical disabilities that affect an older individual’s possibility to remain independent in daily life may have been addressed by supportive measures provided by a nursing home or through home care, however, we did not have information on this.

We also identified several sociodemographic and health factors associated with the access to specialized rehabilitation. The association with age discussed above, remained after excluding patients who were 65 years or older, suggesting that younger age was positively associated with receiving specialized rehabilitation.^9,22,42^ Males were more likely than females to receive specialized rehabilitation, however, this finding could be due to an older age structure in female TBI patients, since the association did not remain after excluding older individuals (≥65 years), which could explain why previous studies have reported conflicting results.^9,18,22^ Having a current employment or studying was positively associated with accessing specialized rehabilitation, consistent with previous data,^18,19^ even after excluding those aged 65 years or older (ie, the general age of retirement during this time period). Interestingly, Sweden has a tax funded and not insurance dependent healthcare system. Yet, patients closer to the age of retirement and patients without employment were less likely to receive specialized rehabilitation. Specialized rehabilitation may improve independence in daily life and quality of life, and not only to aid return to work or studies.^16,17^ We also found associations between specialized rehabilitation and co-occurring injuries in the grade II and III groups, which could be due to co-occurring injuries being an additional indication of the overall severity of the TBI, which has previously been linked to a higher rate of rehabilitation.^18^

This is to our knowledge, the first study to examine the association with living area and access to rehabilitation after TBI. In-patient rehabilitation facilities are found in all Swedish regions;^36^ however, specialized rehabilitation was less likely to be provided to patients living outside of the cities. This could be due to unawareness, lack of ways of referral in the acute care, or unwillingness from patients to travel far from home. Sweden is a large, scarcely populated country with one of the lowest population densities in Europe,^43^ and the living area could be an important factor to consider when organizing post-TBI rehabilitation, both when identifying possible biases in referral to specialized rehabilitation, and when providing new methods of rehabilitation. Telerehabilitation has emerged rapidly during the COVID pandemic and could be a promising way of providing access to specialized rehabilitation after TBI regardless of where the patient lives.^44^

In line with previous studies,^45^ we found high prevalence rates of diagnosed substance use disorder amongst patients with grade I TBI (18%). Substance abuse may increase the risk of sustaining a TBI, as well as hamper recovery following the TBI and the ability to partake in the rehabilitation process.^45^ Our results showed that substance use disorders were negatively associated with receiving specialized rehabilitation in the sensitivity analysis for patients <65 years. Patients could benefit if a current substance abuse is identified and taken into account when individualizing the rehabilitation measures. A multidisciplinary approach with experience of not only the brain injury, but also addiction, could be needed to provide rehabilitation for these patients.

This large nationwide study is without enrollment bias, and data were based on validated high-quality registers,^29,30,46^ where attrition was minimal. Limitations of the current study include that information regarding the type and intensity of rehabilitation was not available and we did not have access to data on non-specialized rehabilitation services provided by primary care or on rehabilitation provided by domestic care and nursing homes. We lacked detailed clinical data for the classification of TBI severity (such as the Glasgow Coma Scale),^47^ but we used other proxies for severity of injury, such as the combination of TBI diagnoses and neurosurgical procedure codes, which has shown high sensitivity and specificity for severe TBI.^32^ We also used the length of hospital stay as a proxy, which has been associated with the severity of the TBI.^48^ We did not have information on the reason for receiving geriatric care, whether this was due to the TBI diagnosis or any of the comorbidities. Finally, we could not include patients from the last 10 years in this study due to data availability. However, no fundamental reorganization or policy has been adapted in Sweden during this period regarding the pathways of post-TBI care and rehabilitation,^35,49^ and results should thus be generalizable to more recent patients. The generalizability of our results to other countries could be limited by differing organization and structure of healthcare systems and rehabilitation services across countries. However, countries without national guidelines and established pathways for post-TBI rehabilitation may still have similar inequalities due to sociodemographic factors and age.

In conclusion, access to specialized rehabilitation for patients hospitalized for TBI was highest for the most severe patients but lower than could be expected for all severities. The largest group of TBI patients today are of older age, and our study showed that the majority of older patients did not receive rehabilitation by caregivers with experience of brain injury (such as specialized rehabilitation). Additionally, we found that access to specialized rehabilitation or geriatric care was associated with the living area of the patient and employment status. Our results highlight the need of forming and implementing national guidelines where the patient’s needs of rehabilitation are met, regardless of their age, socioeconomic status, health, or living area. Beyond this, future studies should aim to consistently evaluate rehabilitative measures in order to ensure improved outcomes.

## Supplemental Material

sj-docx-3-nnr-10.1177_15459683231209315 – Supplemental material for Access to Rehabilitation After Hospitalization for Traumatic Brain Injury: A National Longitudinal Cohort Study in Sweden Click here for additional data file. Supplemental material, sj-docx-3-nnr-10.1177_15459683231209315 for Access to Rehabilitation After Hospitalization for Traumatic Brain Injury: A National Longitudinal Cohort Study in Sweden by Andrea Klang, Yasmina Molero, Paul Lichtenstein, Henrik Larsson, Brian Matthew D’Onofrio, Niklas Marklund, Christian Oldenburg, and Elham Rostami in Neurorehabilitation and Neural Repair

sj-pdf-1-nnr-10.1177_15459683231209315 – Supplemental material for Access to Rehabilitation After Hospitalization for Traumatic Brain Injury: A National Longitudinal Cohort Study in Sweden Click here for additional data file. Supplemental material, sj-pdf-1-nnr-10.1177_15459683231209315 for Access to Rehabilitation After Hospitalization for Traumatic Brain Injury: A National Longitudinal Cohort Study in Sweden by Andrea Klang, Yasmina Molero, Paul Lichtenstein, Henrik Larsson, Brian Matthew D’Onofrio, Niklas Marklund, Christian Oldenburg, and Elham Rostami in Neurorehabilitation and Neural Repair

sj-pdf-2-nnr-10.1177_15459683231209315 – Supplemental material for Access to Rehabilitation After Hospitalization for Traumatic Brain Injury: A National Longitudinal Cohort Study in Sweden Click here for additional data file. Supplemental material, sj-pdf-2-nnr-10.1177_15459683231209315 for Access to Rehabilitation After Hospitalization for Traumatic Brain Injury: A National Longitudinal Cohort Study in Sweden by Andrea Klang, Yasmina Molero, Paul Lichtenstein, Henrik Larsson, Brian Matthew D’Onofrio, Niklas Marklund, Christian Oldenburg, and Elham Rostami in Neurorehabilitation and Neural Repair
