# Supplementary material for: Access to Rehabilitation After Hospitalization for Traumatic Brain Injury: A National Longitudinal Cohort Study in Sweden
Source: Neurorehabil Neural Repair. 2023 Nov 12;37(11-12):763–74. doi: 10.1177/15459683231209315 (PMC10685696; doi:10.1177/15459683231209315)
Supplement: sj-docx-3-nnr-10.1177_15459683231209315 – Supplemental material for Access to Rehabilitation After Hospitalization for Traumatic Brain Injury: A National Longitudinal Cohort Study in Sweden [file sj-docx-3-nnr-10.1177_15459683231209315.docx]

***Appendix***

[Degree of urbanization (DEGURBA). 1](#_Toc144141774)

[Premorbidities 1](#_Toc144141775)

[Definition of co-occurring injuries 1](#_Toc144141776)

[Appendix Table A. Initial TBI treatment and time of hospital stay 3](#_Toc144141777)

[Appendix Table B. Relative risk ratio for receiving specialized rehabilitation for patients <65 years [CI 95%] (n=5,441) 4](#_Toc144141778)

[STROBE Statement—Checklist of items that should be included in reports of cohort studies^3^ 5](#_Toc144141779)

[References 7](#_Toc144141780)

## Degree of urbanization (DEGURBA).

DEGURBA classifies a municipal in three categories depending on the density of the population. Cities include a majority of highly densely populated areas (>1,500 inhabitants per km^2^), towns and suburban areas have clusters of moderately dense populations (>300 inhabitants per km^2^), and rural areas include a majority of low-density clusters (<300 inhabitants per km^2^). The municipals in Sweden are categorized according to this classification by Statistics of Sweden^1^.

## Premorbidities

Information on lifetime prevalence of neurological and psychiatric disorders before the date of the TBI was collected from the National Patient Register^2^, and included dementia (ICD-10: F00-F03), other neurodegenerative disorders (ICD-10: G10-G37), stroke (ICD-10: I60-63), substance use disorders (ICD-10: F10-F16, F18-F19), and other psychiatric disorders (ICD-10: F20-F99).

## Definition of co-occurring injuries

Co-occurring injuries were defined by the following ICD-10 codes during the initial hospital stay:

Spinal cord injury was defined as an injury to the spinal cord and/or nerve root by the following ICD-10 codes: S14.0-S14.2, S24.0-S24.2, S34.0-S34.3, T06.1, T09.3.

Fracture was defined by the following ICD-10 codes: ICD-10: S12, S22, S32, S42, S52, S62, S72, S82, S92, T02, T12.

Other injury was injuries excluding fractures and spinal cord injuries and TBI diagnoses as above, and defined by the following ICD-10 codes: S00-S99, T00-T19 (excluding the previous specified codes for fractures, spinal cord injuries and TBI).

**Grade I TBI Grade II TBI Grade III TBI**

**(n=1,399) (n=6,657) (n=11,422)**

*Total time of treatment*

Median time of stay* (IQR) 25 (11, 54) 16 (12, 25) 5 (3, 7)

*Treatment by departments*^†^

Neurosurgical department 89.2% (1,218) 25.0% (1,305) 17.2% (1,593)

Median time of stay* (IQR) 10 (5, 18) 5 (3, 10) 3 (3, 5)

Non-neurosurgical department^‡^ 81.1% (1,108) 83.9% (4,385) 83.2% (7721)

Median time of stay* (IQR) 7 (2, 18) 11 (6, 16) 4 (3, 6)

Orthopedic department 2.8% (38) 5.1% (268) 3.8% (348)

Median time of stay* (IQR) 5.5 (1, 14) 10 (5, 15) 4 (2, 6)

Geriatric department 11.0% (150) 25.0% (1,305) 21.8% (405)

Median time of stay* (IQR) 23 (13, 37) 14 (10, 21) 6 (4, 7)

Rehabilitation medicine department 27.6% (377) 9.3% (484) 0.6% (54)

Median time of stay* (IQR) 33 (15, 61) 22 (13, 40) 4 (0, 6)

Other department 13.2% (180) 16.6% (865) 14.1% (1,312)

Median time of stay* (IQR) 0 (0, 7) 8 (0, 14) 2 (0, 4)

* Number of days

^†^ Treatment at the departments is not mutually exclusive (i.e., a patient can have been treated at several departments during the hospital stay)

^‡^ Non-neurosurgical department with experience of neurological monitoring (i.e., surgery, internal medicine, neurology, and stroke)

Grade I = neurosurgical intervention, grade II = >10 days hospitalization, grade III = 3-10 days hospitalization.

## Appendix Table A. Initial TBI treatment and time of hospital stay

## Appendix Table B. Relative risk ratio for receiving specialized rehabilitation for patients <65 years [CI 95%] (n=5,441)

**Specialized rehabilitation for patients <65y**

*Unadjusted^1^*

Grade I 4.84 [4.36,5.38]

Grade II 3.67 [3.29,4.1]

*Adjusted for sex and age^1^*

Grade I 4.66 [4.19,5.18]

Grade II 3.77 [3.38,4.2]

*Full adjustment^1^*^,2^

Grade I 4.68 [4.22,5.2]

Grade II 3.76 [3.38,4.19]

1. Reference Grade III TBI

2. Adjusted for sex, age, degree of urbanization, level of education, source of income, co-occuring injuries and premorbid: substance use disorder, psychiatric illness, neurodegenerative disease and stroke

Grade I = neurosurgical intervention, grade II = >10 days hospitalization, grade III = 3-10 days hospitalization.

## STROBE Statement—Checklist of items that should be included in reports of cohort studies^3^

|  | Item No | Recommendation | Page  No. |
| --- | --- | --- | --- |
| **Title and abstract** | 1 | (*a*) Indicate the study’s design with a commonly used term in the title or the abstract | Title + Abstract p. 1 |
|  |  | (*b*) Provide in the abstract an informative and balanced summary of what was done and what was found | Abstract pp. 1-2 |
| Introduction | | |  |
| Background/rationale | 2 | Explain the scientific background and rationale for the investigation being reported | pp. 3-4 |
| Objectives | 3 | State specific objectives, including any prespecified hypotheses | p. 4 |
| Methods | | |  |
| Study design | 4 | Present key elements of study design early in the paper | Pp. 5-7 |
| Setting | 5 | Describe the setting, locations, and relevant dates, including periods of recruitment, exposure, follow-up, and data collection | pp. 5-6 |
| Participants | 6 | (*a*) Give the eligibility criteria, and the sources and methods of selection of participants. Describe methods of follow-up | pp. 5-9 |
|  |  | (*b*) For matched studies, give matching criteria and number of exposed and unexposed | n/a (cohort study) |
| Variables | 7 | Clearly define all outcomes, exposures, predictors, potential confounders, and effect modifiers. Give diagnostic criteria, if applicable | pp. 5-8 + appendix |
| Data sources/ measurement | 8* | For each variable of interest, give sources of data and details of methods of assessment (measurement). Describe comparability of assessment methods if there is more than one group | pp. 5-9 |
| Bias | 9 | Describe any efforts to address potential sources of bias | pp. 5-9 |
| Study size | 10 | Explain how the study size was arrived at | p. 5 |
| Quantitative variables | 11 | Explain how quantitative variables were handled in the analyses. If applicable, describe which groupings were chosen and why | pp. 7-9 |
| Statistical methods | 12 | (*a*) Describe all statistical methods, including those used to control for confounding | pp. 7-9 |
|  |  | (*b*) Describe any methods used to examine subgroups and interactions | pp. 7*-9* |
|  |  | (*c*) Explain how missing data were addressed | n/a (register data with nationwide coverage) |
|  |  | (*d*) If applicable, explain how loss to follow-up was addressed | n/a (register data with nationwide coverage) |
|  |  | (*e*) Describe any sensitivity analyses | pp. 8-9 |
| Results | | |  |
| Participants | 13* | (a) Report numbers of individuals at each stage of study—eg numbers potentially eligible, examined for eligibility, confirmed eligible, included in the study, completing follow-up, and analysed | p. 5 |
|  |  | (b) Give reasons for non-participation at each stage | p. 5 |
|  |  | (c) Consider use of a flow diagram | App. Fig. A |
| Descriptive data | 14* | (a) Give characteristics of study participants (eg demographic, clinical, social) and information on exposures and potential confounders | pp. 10-11 + table 1 |
|  |  | (b) Indicate number of participants with missing data for each variable of interest | Table 1 |
|  |  | (c) Summarise follow-up time (eg, average and total amount) | p. 10 |
| Outcome data | 15* | Report numbers of outcome events or summary measures over time | pp. 10-13, Fig. 1+2 |
| Main results | 16 | (*a*) Give unadjusted estimates and, if applicable, confounder-adjusted estimates and their precision (eg, 95% confidence interval). Make clear which confounders were adjusted for and why they were included | pp. 10-13, Fig 3 + 4  App. Fig. B1-3 |
|  |  | (*b*) Report category boundaries when continuous variables were categorized | n/a |
|  |  | (*c*) If relevant, consider translating estimates of relative risk into absolute risk for a meaningful time period | n/a |
| Other analyses | 17 | Report other analyses done—eg analyses of subgroups and interactions, and sensitivity analyses | pp. 12-13, App. Fig. B1-3 |
| Discussion | | |  |
| Key results | 18 | Summarise key results with reference to study objectives | p. 14 |
| Limitations | 19 | Discuss limitations of the study, taking into account sources of potential bias or imprecision. Discuss both direction and magnitude of any potential bias | pp. 14-17 |
| Interpretation | 20 | Give a cautious overall interpretation of results considering objectives, limitations, multiplicity of analyses, results from similar studies, and other relevant evidence | pp. 14-17 |
| Generalisability | 21 | Discuss the generalisability (external validity) of the study results | p. 17 |
| Other information | | |  |
| Funding | 22 | Give the source of funding and the role of the funders for the present study and, if applicable, for the original study on which the present article is based | p. 18 |

*Give information separately for exposed and unexposed groups.

**Note:** An Explanation and Elaboration article discusses each checklist item and gives methodological background and published examples of transparent reporting. The STROBE checklist is best used in conjunction with this article (freely available on the Web sites of PLoS Medicine at http://www.plosmedicine.org/, Annals of Internal Medicine at http://www.annals.org/, and Epidemiology at http://www.epidem.com/). Information on the STROBE Initiative is available at http://www.strobe-statement.org.

## References

1. SCB - Swedish Agency for Health Technology Assessment and Assessment of Social Services. *Total Population in Sweden between 1960-2021*. Accessed December 15, 2022. https://www.statistikdatabasen.scb.se/pxweb/sv/ssd/START__BE__BE0101__BE0101A/BefolkningNy/

2. Ludvigsson JF, Andersson E, Ekbom A, et al. External review and validation of the Swedish national inpatient register. *BMC Public Health*. 2011;11. doi:10.1186/1471-2458-11-450

3. von Elm E, Altman DG, Egger M, et al. The Strengthening the Reporting of Observational Studies in Epidemiology (STROBE) statement: guidelines for reporting observational studies. *J Clin Epidemiol*. 2008;61(4):344-349. doi:10.1016/j.jclinepi.2007.11.008
