# Supplementary material for: Access to Rehabilitation After Hospitalization for Traumatic Brain Injury: A National Longitudinal Cohort Study in Sweden
Source: Neurorehabil Neural Repair. 2023 Nov 12;37(11-12):763–74. doi: 10.1177/15459683231209315 (PMC10685696; doi:10.1177/15459683231209315)
Supplement: sj-pdf-1-nnr-10.1177_15459683231209315 – Supplemental material for Access to Rehabilitation After Hospitalization for Traumatic Brain Injury: A National Longitudinal Cohort Study in Sweden [file sj-pdf-1-nnr-10.1177_15459683231209315.pdf]

Figure A. Flowchart of TBI cohort inclusion.

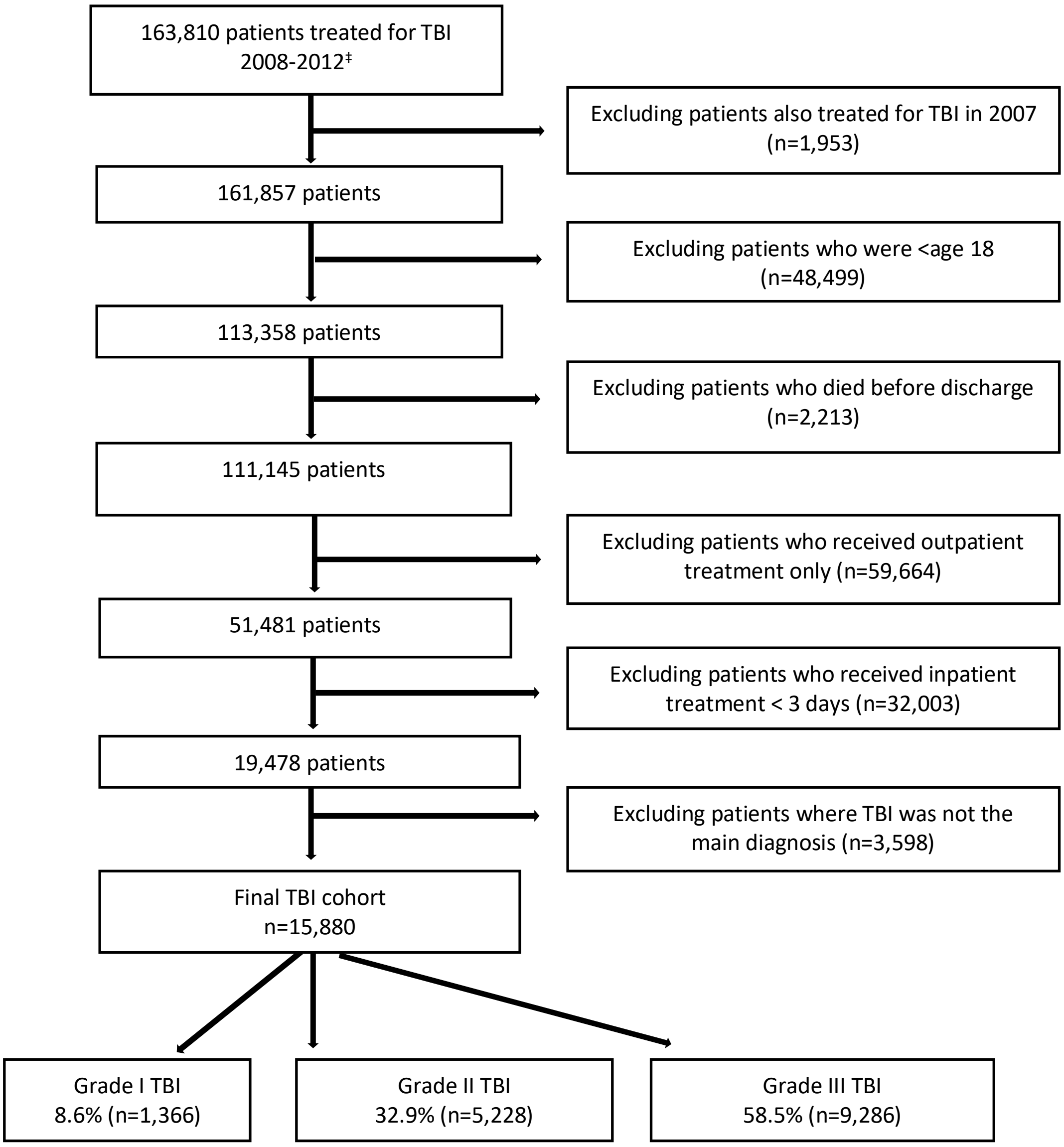

‡Treatment initiated on or after January 1, 2008 and discharge no later than December 31, 2012.
