# Supplementary material for: Access to Rehabilitation After Hospitalization for Traumatic Brain Injury: A National Longitudinal Cohort Study in Sweden
Source: Neurorehabil Neural Repair. 2023 Nov 12;37(11-12):763–74. doi: 10.1177/15459683231209315 (PMC10685696; doi:10.1177/15459683231209315)
Supplement: sj-pdf-2-nnr-10.1177_15459683231209315 – Supplemental material for Access to Rehabilitation After Hospitalization for Traumatic Brain Injury: A National Longitudinal Cohort Study in Sweden [file sj-pdf-2-nnr-10.1177_15459683231209315.pdf]

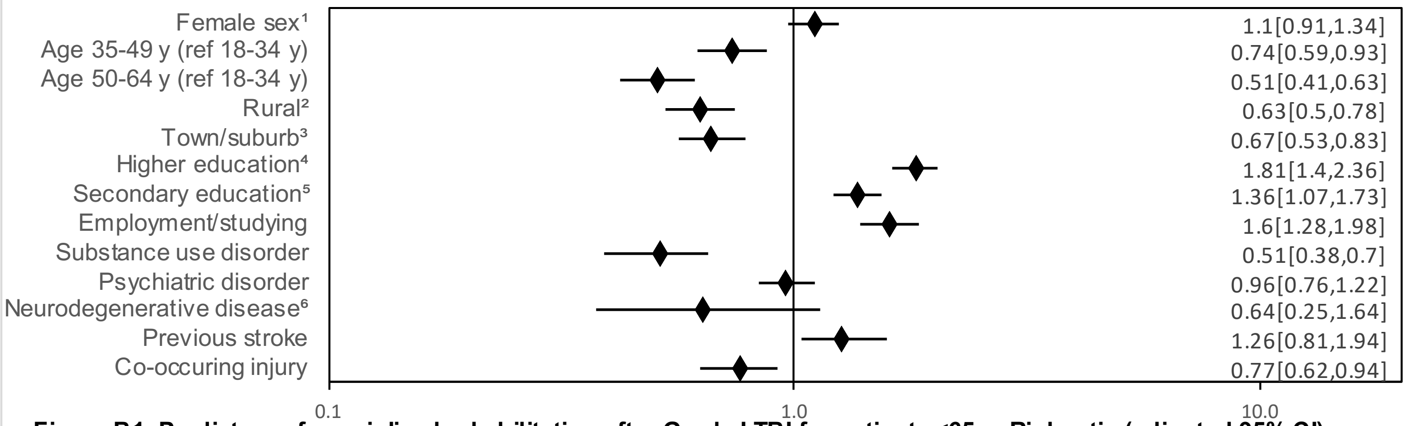

Figure B1. Predictors of specialized rehabilitation after Grade I TBI for patients <65 y. Risk ratio (adjusted 95% CI)

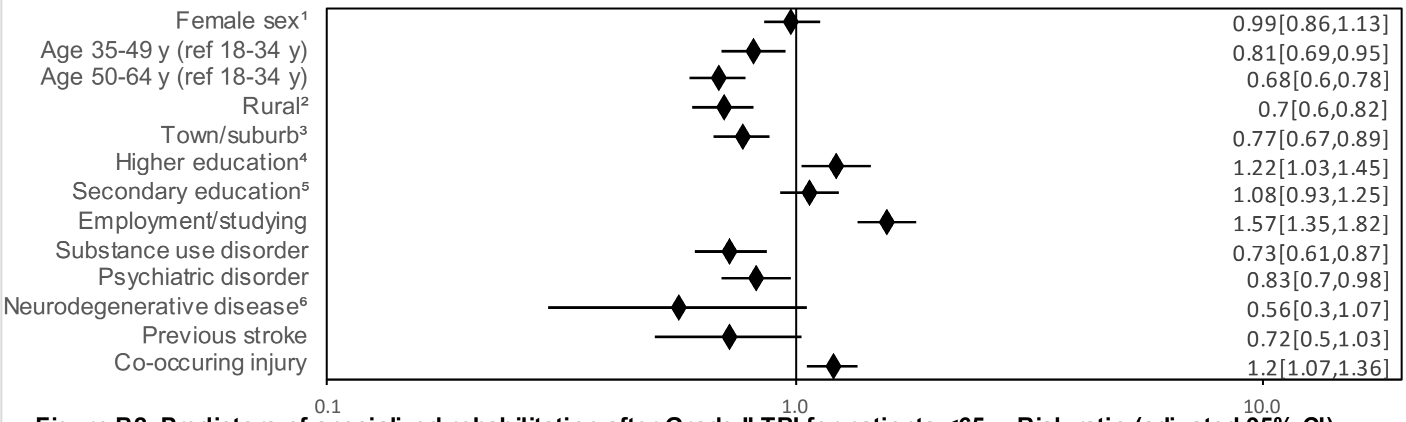

Figure B2. Predictors of specialized rehabilitation after Grade II TBI for patients <65 y. Risk ratio (adjusted 95% CI)

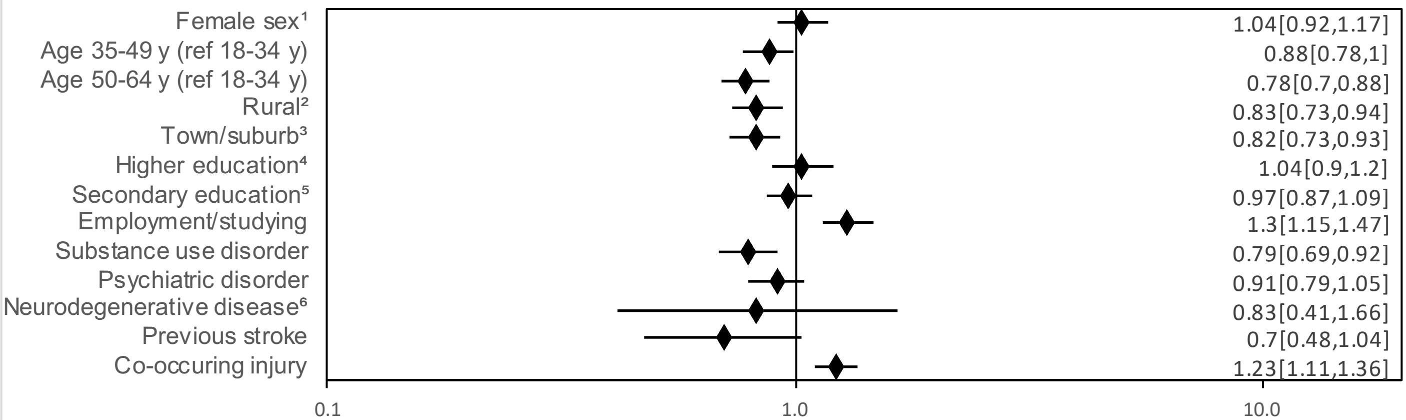

Figure B3. Predictors of specialized rehabilitation after Grade III TBI for patients <65 y. Risk ratio (adjusted 95% CI)

1. Reference male sex.
2. Living in rural area (DEGURBA). Reference City area.
3. Living in town/suburban area (DEGURBA). Reference City area.
4. >12 y of education. Reference primary education <9 y.
5. 10-12 y of education. Reference primary education <9 y.
6. Including dementia

Missing information for the total TBI-cohort: DEGURBA for 62 individuals, level of education for 56 individuals, occupation for 347 individuals.

Grade I = neurosurgical intervention, grade II = >10 days hospitalization, grade III = 3-10 days hospitalization.
